# Predictors of high dose of massed practice following stroke

**DOI:** 10.1515/tnsci-2022-0228

**Published:** 2022-07-14

**Authors:** Bishir Sabo, Auwal Abdullahi, Umaru Muhammad Badaru, Wim Saeys, Steven Truijen

**Affiliations:** Department of Physiotherapy, Bayero University Kano, 70001 Kano, Nigeria; Department of Physiotherapy, Ahmadu Bello University Teaching Hospital, Zaria, Nigeria; Department of Rehabilitation Sciences and Physiotherapy, University of Antwerp, Movant, Wilrijk, Belgium

**Keywords:** dose, motor recovery, activities of daily living, quality of life

## Abstract

**Objective:**

The aim of this study is to determine the factors that affect patients’ ability to carry out high dose of massed practice.

**Methods:**

Patients with stroke were included in the study if they had no severe impairment in motor and cognitive functions. Dose of massed practice, motor function, perceived amount and quality of use of the arm in the real world, wrist and elbow flexors spasticity, dominant hand stroke, presence of shoulder pain, and central post-stroke pain were assessed on the first day. Dose of massed practice was assessed again on the second day. The data were analyzed using descriptive statistics and linear multiple regression.

**Results:**

Only motor function (*β* **=** –0.310, *r* = 0.787, *P* < 0.001), perceived amount of use (*β* **=** 0.300, *r* = 0.823; 95% CI = 0.34–107.224, *P* = 0.049), severity of shoulder pain (*β* **=** –0.155, *r* = –0.472, *P* = 0.019), wrist flexors spasticity (*β* **=** –0.154, *r* = –0.421, *P* = 0.002), age (*β* **=** –0.129, *r* = –0.366, *P* = 0.018), dominant hand stroke (*β* **=** –0.091, *r* = –0.075, *P* = 0.041), and sex (*β* **=** –0.090, *r* = –0.161, *P* = 0.036) significantly influenced patients’ ability to carry out high dose of massed practice.

**Conclusion:**

Many factors affect patients’ ability to carry out high dose of massed practice. Understanding these factors can help in designing appropriate rehabilitation.

## 1 Introduction

Good function of the motor system is essential for carrying out activities of daily living (ADL) effectively. For instance, moving one’s upper limb is essential for eating, buttoning one’s shirt, breast feeding a baby, opening the door, bathing, cooking, and wearing shoes or clothes. However, the function of the motor system which is essential for movements that allow us to carry out our ADL may be impaired following stroke [1]. Consequently, people with stroke may not be able to use the affected limb in carrying out ADL. In addition, ADL such as bathing, cooking, and buttoning one’s shirts that require the use of both hands may not be carried out effectively. Therefore, rehabilitation to improve movement is important to regain the aforementioned functions. This can be achieved through the use of a number of rehabilitation techniques such as the constraint-induced movement therapy (CIMT) [2–5].

The CIMT is a neurorehabilitation technique used to improve functions of the motor system in people with disorders of the brain, particularly stroke [5–7]. The technique has many components; however, the chief among them include massed practice, constraint, and transfer package [8]. The massed practice involves repetitive practice of functional tasks with the affected limb. It is being regarded as the main driver for recovery, as it is essential for inducing use-dependent plasticity [9,10]. The constraint involves restraint of the unaffected limb with a mitt or sling for hours during the day to help maximize the use of the affected limb in the real world or laboratory or clinic. However, it is important to note that, the constraint does not have to be physical. Behaviural constraint wherein the patients consciously limit the use of the unaffected limb is also used [11,12]. The transfer package involves a contract that is designed for the patient to use the affected limb in daily activities in the real world in order to maximize recovery of function [8].

The neuroscientific basis for these three major components of CIMT (massed practice, constraint, and transfer package) is to help reverse learned non-use phenomenon that occurs after stroke [13,14]. This is said to be achieved through its influence on molecular activities, anatomical structures, and neurophysiological functions of the brain such as increased expression of brain-derived neurotrophic factor, increased cortical activation, and increased gray matter in sensory and motor areas [15–18]. Consequently, use of the limb in carrying out ADL is improved [5,19]. However, a pre-requisite for massed practice to effect recovery of brain’s functions is that, its dose needs to be as high as practical [9,20]. In essence, high dose of massed practice is required for recovery of motor function. In addition, what is very interesting is that, as long as patients performed high-dose massed practice, it does not matter whether the tasks carried out are specific or non-specific [21]. Furthermore, this high dose of massed practice required for motor recovery has been reported in the literature to range between 300 and 600 repetitions per day [7,22].

Interestingly, Birkenmeier and colleagues reported that, patients with stroke can carry out about 300 repetitions of massed practice within 1 h [22]. This seems to suggest that, high dose of massed practice during CIMT is possible. However, whether the ability to carry out high dose of massed practice is influenced by the clinical and personal characteristics of the patients, seems not to be determined. This is because in the study by Birkenmeier and colleagues, the patients were within chronic stage of stroke and the study participants had moderate movement ability of the upper limb. Therefore, the aim of this study is to determine the personal and clinical characteristics or factors that can influence patients’ ability to carry out high dose of massed practice during CIMT. Similarly, factors that could predict perceived amount and quality of use of the arm in the real world were also investigated.

## 2 Method

### 2.1 Study design

This study is a cross-sectional (observational) study, approved by the Research Ethics Committee of Ahmadu Bello University Teaching Hospital, Zaria, Nigeria (Approval number, 954524802).

### 2.2 Study population

The study population is inpatient and outpatient stroke survivors in Ahmadu Bello University Teaching Hospital (Shika and Tudun Wada sites) in Kaduna state, Nigeria.

The inclusion criteria used for the selection of the study participants are: participants with clinical diagnosis of stroke, who met ICD-9 criteria, with no very severe impairment in motor function as indicated by a score of 1–3 on the motor arm item of the National Institutes of Health Stroke Scale (NIHSS) and a score of three or more on the upper arm item of the motor assessment scale. Additionally, participants were included if they had no severe cognitive impairment as indicated by a score of one or less on the consciousness and communication items of the NIHSS, had ability to perform two-step commands, and had a score of less than eight on the Short Blessed Memory Orientation and Concentration Scale [23,24]. However, participants were excluded if they had neglect indicated by more than three errors on the star cancellation test and sensory loss of two or more points on the sensory item of NIHSS [24].

### 2.3 Sample size estimation

The minimum sample size estimated for the study was 131 patients with stroke. The sample size was estimated (for linear multiple regression) using G-power software [25]. The parameters used for the estimation are effect size *f* ^2^ = 0.15, *P* = 0.05, power = 80% and the number of independent variable = 13 (age, sex, side affected, dominant hand stroke [before stroke], type of stroke, time since stroke, upper limb motor function, perceived amount and quality of the use of the arm in the real world, elbow and wrist flexors spasticity, presence of shoulder pain, and central post-stroke pain [CPSP]). However, 10% attrition rate (13) was added to make the total sample, 144.

The sampling technique used in the study was convenience sampling technique based on the above study inclusion and exclusion criteria.

### 2.4 Data collection procedure

Screening of the study participants for eligibility was carried out by qualified physiotherapists (one in each of the study sites), who were blinded to the aims of the study. For the patients who are eligible for inclusion in the study, their demographic and personal characteristics such as age, sex, time since stroke, and the types of stroke were recorded using a data capture sheet prepared purposely for the study.

Following this, the participants were asked to sit in a chair that has arm rest with a wooden table in front. An empty cup made of plastic with a fixed handle was placed on the table very close to the chair. The participants were made to carry out massed practice by picking up the cup from the table and taking it to their mouths. The massed practice was carried out for 1 h. Picking up a cup from the table and taking it to the mouth was chosen because it is commonly done in the real world. In addition, following stroke, performing tasks such as picking up a cup and taking it to the mouth can be challenging [7]; though, it can be more challenging to some patients compared to others depending on their motor ability.

During the practice, arm slings were worn by the participants in the unaffected upper limbs to ensure constraint of the limb. Stop watch was used to determine the time the participants started and completed performing massed practice. In addition, pen and paper were used to record the number of times (dose) the massed practice was performed by each participant. The massed practice was carried on the first and the second days. Thereafter, the average of the number of times (dose) the massed practice was carried out on the first and second days was calculated for each of the participants. The massed practice was timed using a stop watch.

Other outcomes that were assessed are motor function assessed using Wolf motor function test (WMFT), perceived amount and quality of use of the arm in the real world assessed using motor activity log (MAL), wrist and elbow flexors spasticity assessed using modified Ashworth scale (MAS), dominant hand stroke (before stroke) assessed using Oldfield Handedness Questionnaire, severity of shoulder pain assessed using visual analogue scale (VAS), and CPSP assessed using Douleur Neuropathique 4 Questionnaire (DNQ4). The WMFT consists of 17 items that are scored from zero to five. The higher the score, the better the motor function [3]. The measure has been reported to have good construct and criterion-related validity and inter-rater reliability [26]. The MAL has two parts that measure perceived amount and quality of use of arm in the real world [4]. In total, it consists of 30 items in which each of the items is scored from zero to five. A higher score indicates that, the perceived amount or quality of use of arm in the real world is good. The scale has been reported to be valid and reliable [27,28].

The MAS is a reliable measure of spasticity scored as 0, 1, +1, 2, 3, or 4, with 0 indicating absence of spasticity [29]. However, for the sake of statistical analysis, we considered a score of +1 as 2, a score of 2 as 3, a score of 3 as 4 and a score of 4 as 5. The VAS is a reliable instrument that consists of a 0–10 cm horizontal or vertical line used to assess patients’ report of the severity of their pain, with 0 denoting least pain and 10 denoting highest pain [30,31]. The DNQ4 is a reliable instrument that consists of four items capable of differentiating between neuropathic pain and non-neuropathic pain [32,33]. It is used in the assessment of CPSP. CPSP is a neuropathic pain syndrome following stroke that is characterized by pain and sensory abnormalities in the affected body part [34]. The Oldfield Handedness Questionnaire is a 20 items inventory that is rated by direct observation of the individuals’ behavior to help identify handedness [35]. All the study outcomes were assessed by well trained and blinded assessors (blinded to the aims of the study) who are qualified physiotherapists (one in each of the study sites).

### 2.5 Data analysis procedure

Participants’ demographic characteristics were summarized using descriptive statistics. Linear multiple regression analysis was used to determine which of the independent variables could significantly predict the participants’ ability to carry out high-dose massed practice required for recovery of motor function, and perceived amount and quality of use of the arm in the real world. The level of significance was set at <0.05. All the analyses were performed using SPSS version 20.


**Ethical approval:** The research related to human use has been complied with all the relevant national regulations, institutional policies, and in accordance the tenets of the Helsinki Declaration, and has been approved by the Research Ethics Committee of Ahmadu Bello University Teaching Hospital, Zaria, Nigeria (Approval number, 954524802).
**Informed consent:** Informed consent has been obtained from all individuals included in this study.

## 3 Results

### 3.1 Characteristics of the study participants

**Figure 1:**
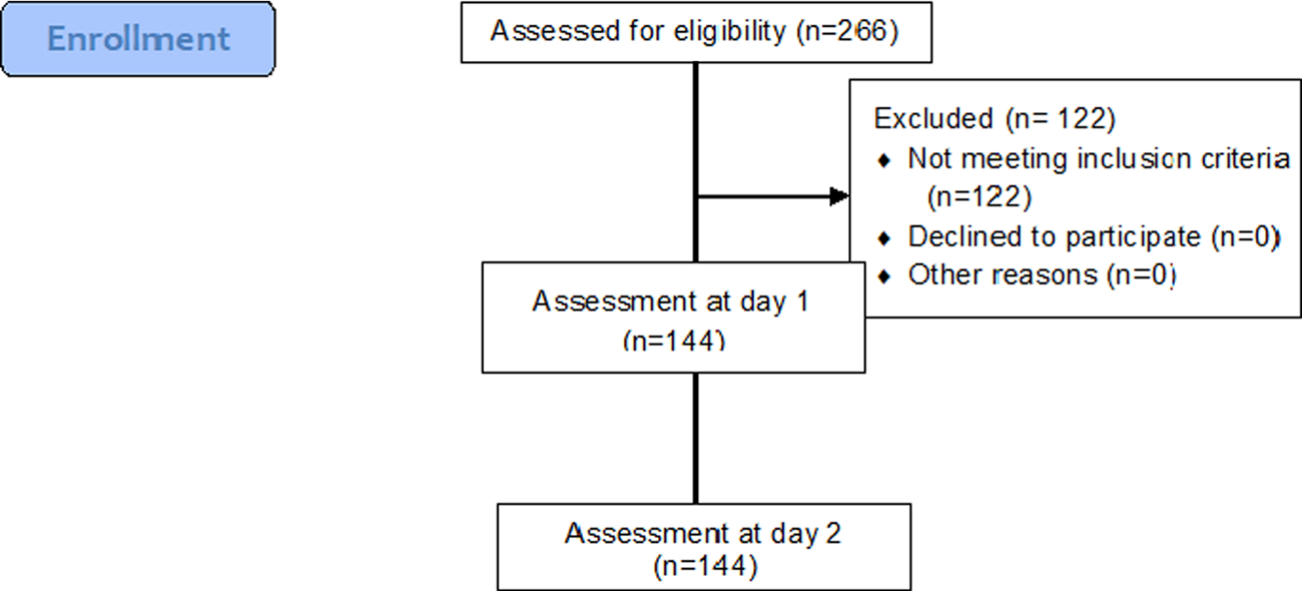
Study flowchart.

One hundred and forty four patients with stroke with age range, 21–101 years and time since stroke range, 1–208 weeks participated in the study. Eighty eight of the participants were men, while 56 were women. Details of the characteristics of the study participants are presented in Table 1. See also Figure 1 for the study flowchart.

**Table 1:** Characteristics of the study participants

Variable	Mean ± SD/Median (Interquartile range)	*n*	%
Sex (male = 1; female = 2)		88/56	61.1/38.9
Type of stroke (ischemic = 1; hemorrhagic = 2)		75/69	52.1/47.9
Dominant hand stroke (before stroke) (right = 1; left = 2)		126/18	87.5/12.5
Side affected (right = 1; left = 2)		101/43	70.1/29.9
Age (years)	58.71 ± 19.90		
Time since stroke (weeks)	36.38 ± 39.99		
Perceived amount of use (MAL [AOU], 0–5)	3.00 ± 0.57		
Perceived quality of use (MAL [QOU], 0–5)	3.05 ± 0.59		
Motor function ([WMFT], 0–5)	1.96 ± 0.74		
Dose of massed practice (number of repetition of the task per hour)	437.50 ± 99.18		
Star cancellation	0.70 ± 0.84		
Star cancellation error	1.00(1.00)		
Number of rest	3.80 ± 1.28		
Severity of shoulder pain ([VAS], 0–10 cm)	1.31 ± 1.32		
Wrist flexors spasticity ([MAS], 0–5)	0.00(0.00)		
Elbow flexors spasticity ([MAS], 0–5)	0.50(1.00)		
CPSP	1.00(2.00)		
Severity of arm paresis	0.50(1.00)		
Severity of sensory loss	0.00(0.00)		
Cognitive ability	2.00(3.50)		

MAL [AOU], motor activity log [amount of use]; MAL [QOU], motor activity log [quality of use]; WMFT, Wolf motor function test; VAS, visual analogue scale; MAS, modified Ashworth scale.

### 3.2 Predictors of ability to carry out high dose of massed practice

The participants performed an average of 437.50 ± 99.18 dose of massed practice with a range 220–634. However, the mean and standard deviation of the residuals for dose of massed practice was 433.14 ± 89.62.

**Figure 2:**
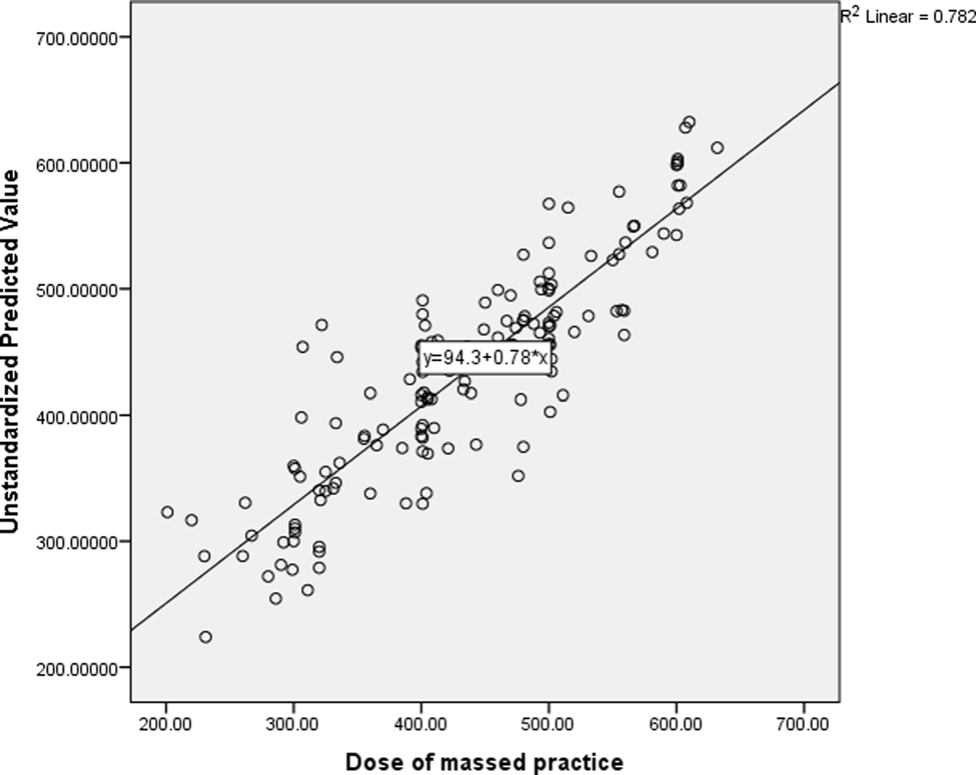
Scatter plot illustrating the regression line of predictors of ability to carry out high dose of massed practice during CIMT.

The result of the linear multiple regression showed that, the total variance explained by the whole model was significant, 88.4% (*R* = 0.884), *F*(13, 144) = 35.931, *R* ^2^ = 0.782, *P* < 0.001. See Figure 2 for the scatter plot illustrating the regression line.

In the final model, the only independent variables that significantly predicted patients’ ability to carry out high dose of massed practice were motor function (*β* = –0.31, *P* < 0.001), perceived amount of use of the arm in the real world (*β* = 0.30, *P* = 0.049), severity of shoulder pain (*β* = –0.16, *P* = 0.019), wrist flexors spasticity (*β* = –0.15, *P* = 0.002), age (*β* = –0.129, *P* = 0.018), dominant hand stroke [before stroke] (*β* = –0.09, *P* = 0.041), and sex (*β* = –0.09, *P* = 0.036). See Table 2 for the details of the result.

**Table 2:** Predictors of high dose of massed practice

Variables	*β*	*r*	95%	*P*
Age (years)	–0.129	–0.366	–1.198 to 0.114	0.018*
Time since stroke (weeks)	–0.049	–0.162	–0.34 to 0.093	0.260
Type of stroke (ischemic = 1; hemiplegic = 2)	–0.004	–0.332	–21.458 to 20.00	0.945
Dominant hand stroke (before stroke) (right = 1; left = 2)	–0.091	–0.075	–54.259 to –1.175	0.041*
Severity of shoulder pain (VAS, 0–10 cm)	–0.155	–0.472	–21.843 to –1.946	0.019*
Wrist flexors spasticity ([MAS], 0–5)	–0.154	–0.421	–52.708 to –11.597	0.002*
Elbow flexors spasticity ([MAS], 0–5)	–0.050	–0.556	–33.432 to 14.897	0.449*
CPSP	0.102	–0.548	–3.639 to 18.931	0.812
Side affected (Right = 1; Left = 2)	–0.041	0.010	–27.876 to 9.936	0.350
Perceived amount of use (MAL [AOU], 0–5]	0.300	0.823	0.34 to 107.224	0.049*
Perceived quality of movement (MAL [QOU], 0–5]	0.132	0.979	–22.831 to 68.102	0.326
Motor function (WMFT, 0–5)	0.310	0.787	19.830 to 64.513	<0.001*
Sex (male=1; female=2)	–0.090	–0.161	–35.862 to –1.268	0.036*

VAS, visual analogue scale; MAS, modified Ashworth scale; MAL [AOU], motor activity log [amount of use]; MAL [QOU], motor activity log [quality of use]; WMFT, Wolf motor function test.

^*^Significance at *p* < 0.05.

### 3.3 Predictors of perceived amount of use of the arm in the real world

The observed mean perceived amount of use of the arm in the real world was 3.05 ± 0.59. However, the mean and standard deviation of the residuals for perceived amount of use of the arm was 3.00 ± 0.50.

**Figure 3:**
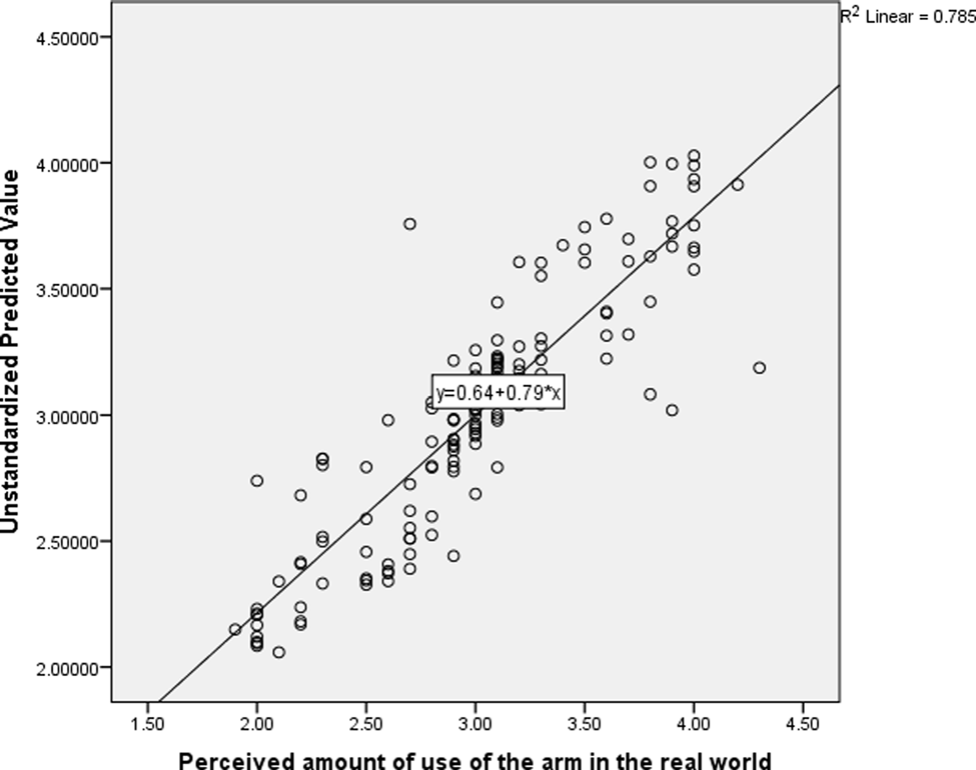
Scatter plot illustrating the regression line of predictors of perceived amount of use of the arm in the real limb following CIMT.

The result of the multiple regression analysis showed that, the total variance explained by the whole model was significant, 88.6% (*R* = 0.886), *F*(11, 144) = 43.875, *R* ^2^ = 0.785, *P* < 0.001. See Figure 3 for the scatter plot illustrating the regression line.

In the final model, the only independent variables that significantly predicted perceived amount of use of the arm in the real world were motor function (*β* = 0.699, *P* < 0.001) and CPSP (*β* = –0.159, *P* = 0.034). See Table 3 for the details of the result.

**Table 3:** Predictors of perceived amount of use of the arm in the real world

Variables	*β*	*r*	95%	*P*
Age (years)	–0.079	–0.275	–0.005 to 0.001	0.131
Time since stroke (weeks)	–0.012	–0.094	–0.001 to 0.001	0.773
Type of stroke (ischemic = 1; hemiplegic = 2)	0.046	–0.295	–0.061 to 0.165	0.364
Dominant hand stroke (before stroke) (right = 1; left = 2)	0.055	0.048	–0.050 to 0.236	0.199
Severity of shoulder pain (VAS, 0–10 cm)	0.029	–0.440	–0.042 to 0.067	0.653
Wrist flexors spasticity ([MAS], 0–5)	–0.092	–0.361	–0.219 to 0.004	0.059
Elbow flexors spasticity ([MAS], 0–5)	–0.110	–0.618	0.245 to 0.015	0.082
CPSP	–0.159	–0.601	–0.127 to –0.005	0.034*
Side affected (right = 1; left = 2)	–0.071	0.064	–0.190 to 0.016	0.097
Motor function (WMFT, 0–5)	0.699	0.850	0.450–0.611	<0.001^*^
Sex (right = 1; left = 2)	0.006	–0.090	0.087–0.102	0.876

VAS, visual analogue scale; MAS, modified Ashworth scale; WMFT, Wolf motor function test.

^*^Significance at *p* < 0.05.

### 3.4 Predictors of perceived quality of use of the arm in the real world

The observed mean perceived quality of use of the arm in the real world was 3.00 ± 0.57. However, the mean and standard deviation of the residuals for perceived quality of use of the arm in the real world was 3.05 ± 0.51.

**Figure 4:**
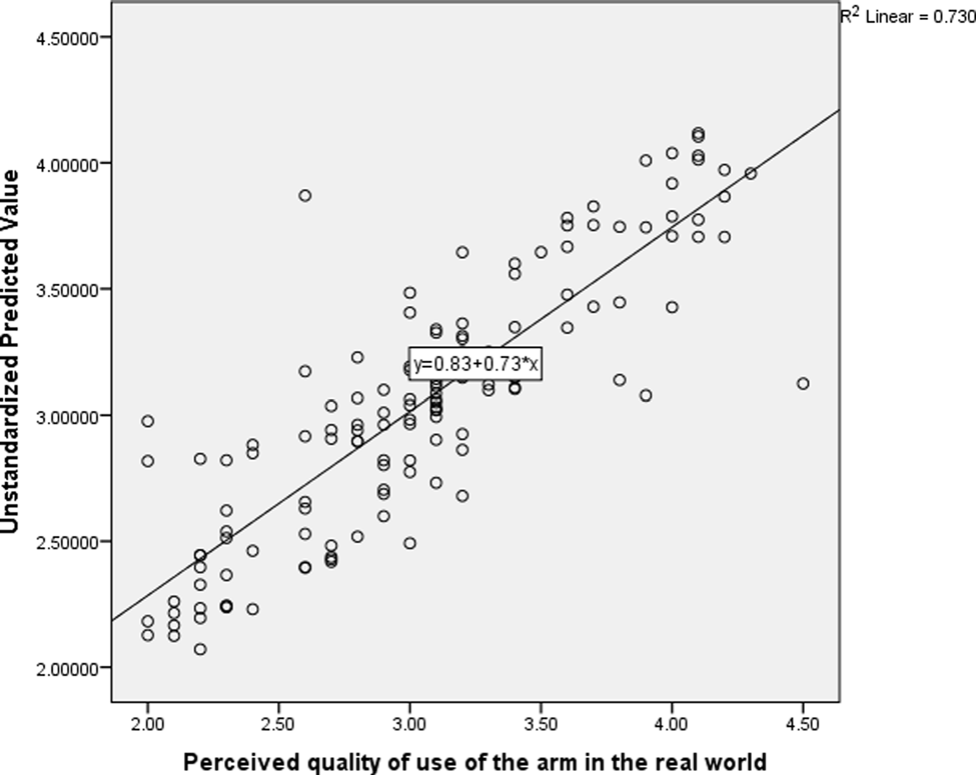
Scatter plot illustrating the regression line of predictors of perceived quality of use of the arm in the real limb following CIMT.

The result of the multiple regression analysis showed that, the total variance explained by the whole model was significant, 85.4% (0.854), *F*(11, 144) = 32.418, *R* ^2^ = 0.73, *P* < 0.001. See Figure 4 for the scatter plot illustrating the regression line.

In the final model, the only independent variables that significantly predicted perceived quality of use of the arm in the real world were motor function (*β* = 0.714, *P* < 0.001) and dominant hand stroke (before stroke) (*β* = 0.105, *P* = 0.029). See Table 4 for the details of the result.

**Table 4:** Predictors of perceived quality of use of the arm in the real world

Variables	*β*	*r*	95%	*P*
Age (years)	–0.109	–0.299	–0.007 to 0.000	0.066
Time since stroke (weeks)	–0.012	–0.019	–0.002 to 0.001	0.695
Type of stroke (ischemic = 1; hemiplegic = 2)	0.068	0.281	–0.053 to 0.213	0.238
Dominant hand stroke (before stroke) (right = 1; left = 2)	0.105	0.083	–0.019 to 0.355	0.029*
Severity of shoulder pain (VAS, 0–10 cm)	–0.022	–0.411	–0.055 to 0.074	0.766
Wrist flexors spasticity ([MAS], 0–5)	–0.082	–0.312	–0.232 to 0.030	0.131
Elbow flexors spasticity ([MAS], 0–5)	–0.026	–0.549	–0.181 to 0.124	0.712
CPSP	–0.156	–0.566	–0.140 to 0.044	0.062
Side affected (right = 1; left = 2)	–0.059	0.066	–0.197 to 0.045	0.215
Motor function (WMFT, 0–5)	0.714	0.822	0.473 to 0.662	<0.001*
Sex (right = 1; left = 2)	0.006	–0.119	0.148 to 0.074	0.513

VAS, visual analogue scale; MAS, modified Ashworth scale; WMFT, Wolf motor function test.

^*^Significance at *p* < 0.05.

## 4 Discussion

The result showed that, only seven independent variables (motor function, perceived amount of use of the arm in the real world, severity of shoulder pain, wrist flexors spasticity, age, dominant hand stroke, and sex) significantly influenced the participants’ ability to carry out high dose of massed practice per day, required for the recovery of motor function. For the real world arm use, only motor function and CPSP; and motor function and dominant hand stroke significantly influenced perceived amount and quality of use of the arm, respectively. However, the mean scores for motor function and perceived amount of use of the arm indicate mild motor ability and moderate perceived amount of use of the arm, respectively. In addition, severity of shoulder pain was mild, there was little or no wrist flexors spasticity, the patients were middle aged, most of the participants had dominant hand stroke and the majority were men. Good motor function is the evidence for the integrity of the motor system that controls human movement. Ability to move is strongly related to the ability to carry out ADL [36].

Similarly, ability to carry out ADL such as washing, bathing, feeding, cutting meat or a loaf of bread, and walking is also a predictor of good quality of life [37]. Achieving good quality of life is the ultimate goal of rehabilitation. Furthermore, there is a strong positive correlation between how frequently the arm is used in carrying out tasks in the real world (perceived amount of use of the arm) and motor function [38]. Therefore, it is important to encourage people with stroke to use their affected arms in the real world. This is the rationale for transfer package, one of the most important components of CIMT. Transfer package is a set of behavioral techniques used to help patients transfer the therapeutic gains following CIMT to real daily life situations [39]. Thus, CIMT protocols should include transfer package and tasks that are related to the patient’s everyday tasks that consider their cultural practices or norms.

Presence of pain especially pain in the shoulder during the first and third months after the stroke can limit movement [40]. Therefore, pain management especially shoulder pain should be a priority in order to help patients with stroke carry out high-dose massed practice during CIMT. In addition, it is important for the therapists to determine the dose of massed practice patients with pain in the arm can carry out during CIMT. Accordingly, in a child with severe shoulder pain and motor impairment following cerebral malaria, 75 repetitions of massed practice per day for 6 weeks resulted in marked improvement in motor function [41]. This can serve as a model for the dose of massed practice to be used in people with stroke who have pain that could limit their ability to carry out high dose of massed practice.

Spasticity can affect movement pattern [42], and this can slow down movement speed and efficiency. Consequently, this may have downstream effects on the patients’ quality of life [43]. Therefore, in patients with wrist and/or elbow flexors spasticity, it is important to ask patients to carry out a specific dose of massed practice in terms of how many times the practice is done rather than asking them to carry it out in for example 2 h. This is because the patients may not be able to achieve the dose of massed practice required for improvement within those recommended hours. In addition, spasticity may be associated with pain [44]. Both pain and spasticity can limit function. As such, for patients to be able to carry out high dose of massed practice, spasticity and pain as the case may be, need to be managed promptly. Furthermore, as we chronologically age, the structures of the nervous system (both the central and peripheral nervous systems) age too [45]. This may lead to motor performance deficits such as impaired coordination and speed [46].

In addition, older people may suffer fatigue quite readily compared to younger people. All these can hinder the patients’ ability to carry out high dose of massed practice. However, use of distributed tasks practice in which practice is done in sessions per day to help with preventing the effects of fatigue, can help older patients to achieve high-dose massed practice required for recovery. Distributed practice has been used with success [47].

For sex influence, women tend to perform daily tasks less than men after stroke [48,49]. However, this can be attributed to old age and lower pre-stroke physical function level [48]. Similarly, it may also be related to loss of internal locus of control. Thus, in designing CIMT protocol for female patients, measures such as motivational interviewing should be used to motivate them. Such measures of motivation have been used to improve stroke patients’ performance in physical function [50]. In addition, women tend to be more depressed and have anxiety more than men after stroke [49]. Depressive symptoms are negatively correlated with all variables of motor skills [50,51]. Therefore, it is important to improve the mental health of patients especially women undergoing CIMT. This will enable them achieve the desired goal.

Individuals with dominant hand stroke demonstrate less impairment than those with non-dominant hand stroke [52]. Thus, they may be able to perform high-dose massed practice during CIMT. Massed practice with the dominant hand results in better improvement in motor function [53]. Consequently, therapists should devise means through which patients with non-dominant hand stroke can be able to practice high-dose massed practice. This can be done through the use of distributed practice whereby practice sessions in a day can be spread to suit the patients’ capability. In addition, transfer package, one of the components of CIMT can be used to help such patients achieve high-dose massed practice. Transfer package helps with adherence and extension of therapy beyond the laboratory or clinic. Adherence and increasing dose of therapy improves outcomes [54]. Furthermore, although side affected may influence dose of massed practice because of hemisphere-specific motor deficit [55]; in this study, side affected did not significantly influence dose of massed practice. This is probably because the patients included in the study had mild to moderate impairment in motor function.

Although this study has some strengths such as its relatively large sample size; it however has some key limitations such as lack of follow-up of the participants. This limitation can affect the reliability of the findings, and therefore it needs to be factored in when conducting future studies on the subject matter. In addition, motivation and enriched environment were not assessed in this study. Motivation and enriched environment are some of the factors that can affect recovery of motor function [56].

## 5 Conclusion

Many personal and clinical characteristics of patients with stroke can affect their ability to carry out high-dose massed practice during rehabilitation. Therefore, it is important that these factors are factored in during rehabilitation in order to help patients achieve the dose of massed practice required for recovery following stroke. In particular, distributed practice and transfer package can be used to help patients with stroke achieve high-dose massed practice during CIMT.
